# HIV infection and latency induce a unique metabolic signature in human macrophages

**DOI:** 10.1038/s41598-019-39898-5

**Published:** 2019-03-08

**Authors:** Paul Castellano, Lisa Prevedel, Silvana Valdebenito, Eliseo A. Eugenin

**Affiliations:** 1 Public Health Research Institute (PHRI), Newark, NJ USA; 2 Department of Neuroscience, Cell Biology and Anatomy, University of Texas Medical Branch (UTMB), Galveston, Texas USA

## Abstract

Currently, a major barrier to curing HIV infection is the generation of tissue-associated, non-replicating, long-lasting viral reservoirs that are refractory to therapy and can be reactivated upon anti-retroviral therapy interruption. One of these reservoirs are latently HIV-infected macrophages. Here, we show that HIV infection of macrophages results in survival of a small population of infected cells that are metabolically altered and characterized by mitochondrial fusion, lipid accumulation, and reduced mitochondrial ATP production. No changes in glycolysis were detected. Metabolic analysis indicated an essential role of succinate and other TCA metabolites in the tricarboxylic acid (TCA) cycle in mediating lipid accumulation and oxidative phosphorylation (OXPHOS) in the mitochondria. Furthermore, we show that while uninfected and HIV infected macrophages use fatty acids and glucose as primary sources of energy, surviving HIV infected macrophages also use glutamine/glutamate as a major energy source, and blocking these new sources of energy resulted in the killing of latent HIV infected macrophages. Together, our data provide a new understanding of the formation, properties, and potential novel ways to eliminate macrophage viral reservoirs.

## Introduction

A key feature of HIV infection that has made it virtually impossible to truly cure this disease is the generation of latent viral reservoirs in different tissues. A viral reservoir corresponds to long-lived infected cells, mainly localized in a specific anatomical compartment, where the replication-competent virus can persist for a longer time than the main pool of actively replicating virus^1–4^. One of these reservoirs is HIV infected macrophages, which are terminally differentiated, non-dividing cells derived from circulating monocytes that reside in all tissues^5^. It is widely accepted that in addition to T cells, monocyte/macrophage lineage cells are among the first cells targeted by HIV^6^ and these cells then allow the virus to spread rapidly by transmission to CD4^+^ T cells^7–9^. Furthermore, it was recently demonstrated that macrophages could sustain HIV replication *in vivo* in the absence of T cells, supporting the hypothesis that macrophages are a primary target of HIV and may help transmit the infection to other cell types even in the absence of CD4^+^ T lymphocytes^1,10^.

Although HIV infection kills most CD4^+^ T cells, a small population of HIV infected macrophages survives for extended periods by harboring the virus in cell membrane invaginations that protect virions from antiretroviral treatment (ART) and circulating neutralizing antibodies^11–13^. Under inflammatory conditions, macrophages derived from recently transmigrated monocytes die after few days^14^, whereas microglia, perivascular, and alveolar macrophages can survive for long periods – from weeks to years^15–17^. The properties of mobility, capacity for tissue infiltration, and extended survival have been proposed by several groups to be critical for the role of macrophages in the generation, stability, dissemination, and reactivation of HIV reservoirs. However, the mechanisms by which these latently HIV-infected cells become viral reservoirs and survive for extended periods of time are unknown.

We have characterized the metabolic profile of latently HIV-infected macrophages and identified several unique metabolic features of these cells. First, HIV infection had a profound overall silencing effect on mitochondrial metabolism. Second, in latently infected macrophages the metabolic steps in the tricarboxylic acid (TCA) cycle preceding oxidative phosphorylation (OXPHOS) were compromised, resulting in lipid accumulation, which is typically observed in several tissues and cells in the HIV infected population^18,19^. In addition to fatty acid and glucose, latent HIV-reservoirs relied on glutamine, glutamate, and alpha-ketoglutarate (α-KG) as a major source of energy. Finally, blocking the use of glutamine, glutamate and alpha-ketoglutarate pathways resulted in a significant killing of the latent HIV infected macrophages. These results reveal a unique metabolic signature of HIV infected macrophages that is similar to the observed in aggressive types of brain cancer, especially in glioblastoma^20–22^. We demonstrated that targeting specific metabolic pathways of viral reservoirs is a promising therapeutic approach to eradicate viral reservoirs in HIV infected individuals.

## Results

### HIV infection of macrophages results in massive early apoptosis, the survival of a small population of HIV infected cells, and mitochondrial enlargement

As we previously described, acute HIV infection of human primary astrocytes, microglia, and macrophages results in massive apoptosis; however, a small population of HIV-infected cells survive and become latently infected^23–29^. Despite these findings, HIV infection of macrophages is controversial in a few scientific circles. There are multiple lines of evidence supporting macrophage infection and pathogenesis in an independent manner than HIV replication on T cells^10,23,30,31^. In addition, we recently reported that apoptosis induced by HIV in human macrophages follows an unusual apoptotic pathway with no significant changes in multiple apoptotic proteins, but a significant increase in the protein Bim, probably to block the formation of the apoptosome^23^. Bim, a highly apoptotic protein, is recruited to the mitochondria without resulting in apoptosis^23^, suggesting that in HIV surviving cells, mitochondrial function is compromised. Furthermore, we identify in the current study that Bcl-2, mcl-1, and hsp-70 and-27 are not affected during the entire time course of the infection (Supplemental Fig. 1), supporting the essential role of Bim in the mitochondria, and not other apoptotic proteins, in the context of HIV.

**Figure 1:**
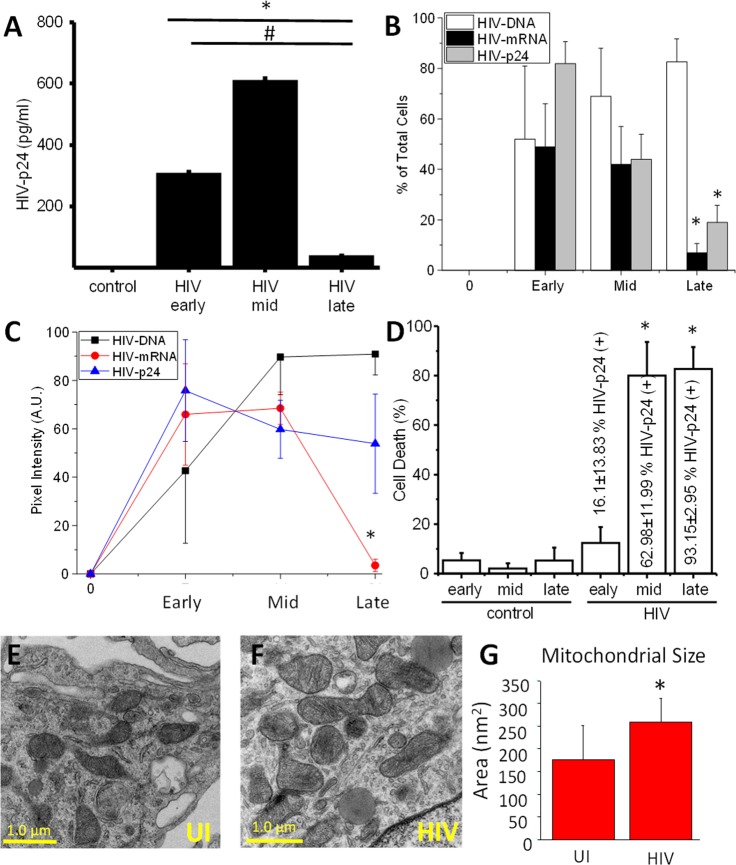
A small population of HIV infected macrophages survives infection and shows mitochondrial compromise. PBMCs were isolated from leukopacks provided by the NY blood center. Macrophages were isolated by adherence in the presence of M-CSF for 7 dpi. Cultures were exposed to 50 ng/ml of HIV_ADA_ for 24 h, washed, and maintained for 3, 7, 14, and 21 dpi for subsequent analysis. (**A**) HIV replication as determined by HIV-p24 ELISA in the supernatant of uninfected (control) and HIV infected macrophage cultures (early, mid, and late). HIV at the late stages becomes silent. HIV DNA staining and Alu-PCR confirmed these results. (**B**) Staining for HIV integrated DNA (nef), viral mRNA (nef) and HIV-p24 protein and subsequent analysis for microscopy. We quantified the % of positive cells at the early, mid, and late stage, indicating that at later time points, viral mRNA and protein expression is decreased in the cells. (**C**) However, quantification of the staining in B, indicates that integrated HIV DNA remains stable, viral mRNA decreased at the late stages, and HIV-p24 remain stable from the mid stages. (**D**) Percentage of cell death in uninfected (control conditions) and HIV infected conditions (HIV). HIV infection induces a large amount of cell death, but early on a population of HIV-p24 positive cells survive the infection. In parenthesis is the % of HIV infected cells. (**E**,**F**) correspond to representative pictures of transmission electron microscopy (TEM) of macrophages in control conditions (uninfected, UI) and HIV infected cells (HIV) to observe the distribution, numbers, and size of mitochondria. (**G**) Quantification of mitochondrial size (nm^2^) per area. *p = 0.0341, n = 7.

As we described, three different stages of viral replication in human macrophages could be observed (Fig. 1A and Supplemental Fig. 2). An early stage (1–3 days post-infection) characterized by increasing HIV replication (Fig. 1 A, HIV early) with ∼50% of the cells with integrated HIV DNA into the host DNA (Fig. 1B, white bars, early). Also at this time point, expression of HIV nef mRNA was detected in ∼50% of the cells (Fig. 1B, early, black bars), and ∼50% of the cells expressed significant amounts of HIV-p24 protein (Fig. 1B, early, gray bars). All these analyses were performed using the simultaneous detection of integrated HIV DNA, viral mRNA, and viral proteins as we recently described in detail^32^. Quantification of the staining for integrated HIV DNA (black line), viral mRNA (red line), and viral proteins (blue line) indicates that HIV-DNA staining remains stable early, mid, and late stages post infection (Fig. 1C). Viral mRNA intensity decreased at the mid-stage indicating a silencing of the viral mRNA expression at the later stages post infection (Fig. 1C). Moreover, the expression of HIV-p24 protein reached a peak at the early stages of infection and remained stable until the later stages post infection, indicating that HIV-p24 protein is stable, despite the decay in HIV mRNA expression (Fig. 1C). All these data together indicate that viral replication becomes silent at the late stages of HIV infection in the few macrophages that survive HIV infection. Quantification of the apoptosis indicates that an early stage (1–3 days post-infection), where 12.36 ± 6.45% of the cells were undergoing apoptosis and 16.1 ± 13.83% of the surviving cells were positive for HIV-p24 (Fig. 1A–D, and^23^). A mid-stage (7–14 days) was characterized by higher viral replication (Fig. 1A), high cell death (79.97 ± 13.65%; Fig. 1C,D), which mainly affected uninfected macrophages (^23^, Fig. 1A–D, “cell death”), and a high percentage of alive cells positive for HIV-p24 (62.98 ± 11.99%; Fig. 1D). The late stages of viral replication (14–21 days) were characterized by minimal to undetectable HIV replication and cell death numbers similar to the mid-stage (82.65 ± 8.94%, Fig. 1D), resulting in the survival of a small population of HIV infected macrophages that were not producing virus (although 93.15 ± 2.95% of the surviving cells were infected. Fig. 1D), also named viral reservoirs (Fig. 1A–C, *p ≤ 0.0021 as compared to uninfected conditions (“control”), ^#^p = 0.0001 as compared to early and mid stages of replication, see representative examples in Supplemental Fig. 2). As expected, no viral replication or unspecific staining was detected in uninfected cells (“control,” Fig. 1A, n = 8).

Analysis of the surviving cells in the HIV-infected cultures by transmission electron microscopy (TEM) demonstrated that mitochondria in the surviving HIV infected cells had become bigger (Fig. 1F) as compared to uninfected cells (Fig. 1E). A similar pattern of mitochondrial enlargement, or fusion, has been observed in different cell types during serum starvation and autophagy conditions^33–35^. Quantification of mitochondrial size demonstrated that mitochondria increased significantly in size after seven days post-infection (Fig. 1G, *p = 0.0341 as compared to uninfected conditions, UI, n = 7). These results were HIV specific and did not replicate using different cell densities or cell activation using cytokines including TNF-α and/or IFN-γ (data not shown). Intriguingly, Western blot and immunofluorescence analysis did not show any significant difference in expression or localization of mitofusin-1 and-2 (Supplemental Fig. 3), as had been described in autophagy^33–35^, indicating that the mechanism of mitochondrial enlargement upon HIV infection is different than in autophagy.

### Mitochondria in surviving HIV infected macrophages are functional

To determine whether the mitochondria in the HIV surviving cells are functional, we measured their relative membrane potential using a ratio between TOM20 and mitotracker. TOM20 staining was not affected by changes in membrane potential (green staining). However, uptake of mitotracker RedCMXRos, which is taken up by a translocase of the mitochondrial outer membrane, is membrane potential-dependent (red staining). We also stained actin using phalloidin staining (white staining) to identify the cell shape. In uninfected cells (UI), as expected, there was a perfect colocalization of TOM20 and mitotracker (Supplemental Fig. 4A). However, in HIV infected cultures, the cells that survived infection showed a loss of colocalization and decreased mitotracker staining (Supplemental Fig. 4A, HIV). Quantification of the TOM20 staining indicated that there was no loss of this protein in uninfected or HIV infected conditions (Supplemental Fig. 4B). Overall, no changes in membrane potential or mitotracker staining were observed among uninfected or HIV infected cells (Supplemental Fig. 4C). However, further analysis of HIV fused cells indicated a reduced mean intensity of mitotracker per cell or per nuclei (Supplemental Fig. 4D) and TOM20 (Supplemental Fig. 4E) (*p = 0.0003, n = 6). Moreover, the ratio of mitotracker/TOM20 remained stable. These data indicate that upon cell to cell fusion due to HIV infection, only a small population of mitochondria was compromised; however, overall mitochondrial potential and numbers were maintained during the time course of infection, especially in surviving HIV infected cells.

### Surviving HIV infected macrophages have reduced overall mitochondrial function but maintain their stress response

To determine whether HIV targets mitochondrial metabolism during different stages of HIV infection of human primary macrophages, we measured their oxygen consumption rate (OCR) using a SeaHorse analyzer (Agilent Technologies, Santa Clara, CA) to determine basal respiration, ATP production, proton leak, maximal respiration, and mitochondrial spare capacity (see details, https://www.agilent.com/en-us/products/cell-analysis-(seahorse)/mitochondrial-respiration-the-xf-cell-mito-stress-test).

**Figure 2:**
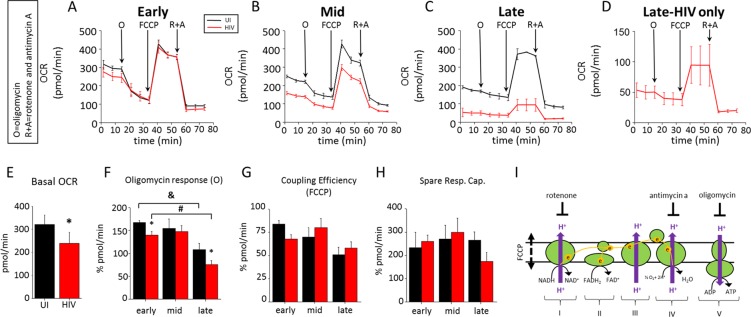
HIV infection reduces the basal OCR in surviving cells. To determine the function of the mitochondria in the population of surviving cells, the OCR (oxygen consumption rate) was determined. Oxygen consumption by complex IV is generally accepted as the main source of oxygen consumption in cells (see the cartoon in I). In these experiments, Complex IV activity can be inhibited by Antimycin A (**A**), and rotenone (R) can shut down complex I activity. Using both inhibitors will completely shut down all ETC activity. Complex V activity can be shut down using oligomycin (O), which will thereby shut down all ATP generated by OXPHOS. The maximal capabilities of mitochondrial ETC function are analyzed by the use of FCCP, an inducer of inner membrane pore formation that dissipates the chemical gradient between the intermembrane space and the matrix. The Seahorse XFp analyzer takes advantage of a sensitive oxygen meter to measure oxygen consumption rates (OCR) in response to the treatments described above. (**A**) Determination of basal respiration, ATP production, maximal respiration and spare capacity using oligomycin, FCCP, and Antimycin A plus rotenone, respectively. Early during infection, there was a reduction in the basal OCR before O treatment. OCR determinations at the mid (**B**) or late stage of infection (**C**). (**D**) shows amplification of the OCR curve during late stages shown in C to correct for the surviving cell number. (**E**) Quantification of the basal OCR (*p = 0.0194, n = 6). (**F**) Quantification of oligomycin response at early, mid and late stages of infection. Only during the early and late stages, where cell death was minimal, were significant differences observed. Red bars correspond to HIV infected cultures (^&^p = 0.0033, ^#^p = 0.00009, n = 3). (**G**) Quantification of coupling efficiency in response to FCCP. (**H**) Quantification of spare respiratory capacity. (**I**) A diagram showing the site of action of each inhibitor.

As indicated in Fig. 2A during the early stage of infection (1–3 days post-infection), the basal OCR was reduced by approximately 20–30%, indicating that early events of HIV infection impacted mitochondrial respiration without changing maximal respiration or spare capacity (Fig. 2A,E). At the mid-stage, where apoptotic levels were high (Fig. 2B,E), we observed a reduction in total OCR that strongly correlated with cell death (Fig. 2B). At the late time points post-infection, where most remaining cells were HIV infected surviving cells, no further changes in the OCR were detected (Fig. 2C,D, amplification of 2 C). Quantification of these data confirmed that HIV infection compromises basal OCR (*p = 0.0194, n = 6), as well as response to oligomycin (Fig. 2F, &, p = 0.0033, n = 3; ^#^p = 0.00009, n = 3), but does not affect FCCP or spare respiratory capacity (Fig. 2G,H, respectively). There was a significant difference in oligomycin response in HIV infected cells compared to uninfected cells early and late during infection, but not during the mid stages, suggesting that during periods of minimal apoptosis, ATP-linked respiration is reduced by the virus (Fig. 2F). These differences in the metabolic profile were not due to the differences in cell number, because subconfluent cultures also have similar behavior to confluent cultures (data not shown). Activation of human macrophages with TNF-α plus IFN-γ did not result in similar metabolic changes (data not shown). Thus metabolic changes are associated with the HIV infection specifically and not due to general immune activation.

### HIV induced mitochondrial compromise is not due to changes in the expression of mitochondrial complexes

Typically, changes in OCR are associated with changes in oxidative phosphorylation (OXPHOS), most specifically complexes I to V^36–38^. To determine whether HIV infection altered the expression of these complexes, qRT-PCR and Western blots were performed. No differences in mRNA expression were found between uninfected, and HIV infected conditions (Supplemental Fig. 5A, n = 7). Results obtained from mRNA amplification of complex V were inconclusive (data not shown). Western blots for complexes I to V indicated no significant differences in expression of all complexes at different stages of infection (Supplemental Fig. 5B,C, n = 5). Thus, HIV-induced changes in mitochondrial shape, basal OCR, and response to oligomycin were not due to changes in mRNA or protein expression related to mitochondrial fusion, as has been described for other diseases^33–35^, indicating that HIV uses a different mechanism to survive infection and/or evade apoptosis.

### Glycolysis is not affected by HIV infection

**Figure 3:**
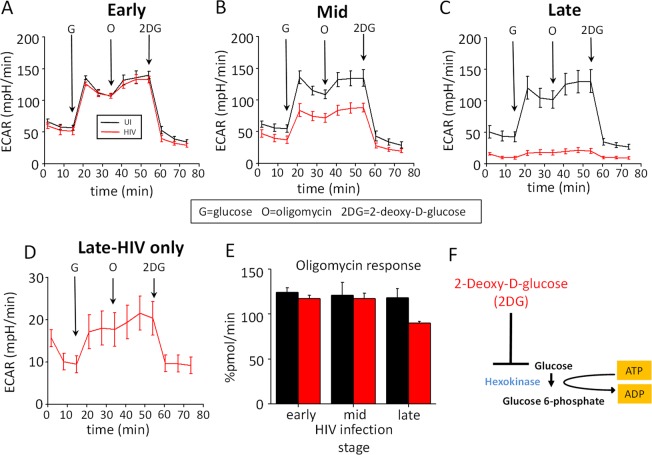
HIV infection did not alter glycolysis. Uninfected (UI) and HIV infected cultures (HIV) at different stages were subjected to ECAR, extracellular acidification rate measurement, and Seahorse analysis to examine glycolysis. We injected glucose (G), oligomycin (O), and 2-deoxy-D-glucose (2DG). Treatment with glucose increases ECAR, while subsequent injection with oligomycin forces cells to use only lactic acid fermentation for ATP production and further increases ECAR. Inhibition with 2DG completely shuts down any ATP production from glycolysis and thus reduces ECAR. (**A**) Changes in ECAR during early stages; (**B**) mid-stages, and (**C**) late stages. (**D**) Corresponds to an amplification of the HIV infected curve shown in C. (**E**) Corresponds to the % change in ECAR in response to oligomycin during all stages of infection. No significant changes were observed. (**F**) Represents the blocking stage of 2DG.

To determine whether the changes in metabolism observed in HIV infected macrophages were due to alterations in glycolysis, extracellular acidification rate (ECAR) was assessed at different time points after HIV infection (i.e., the early, mid and late stages – Fig. 3A–C, respectively). Overall, there were no differences in ECAR in any condition or in response to injection of glucose (G), oligomycin (O), or 2 deoxy-D-glucose (2DG) (see the cartoon in Fig. 3F). These data indicate that glycolysis was not affected by HIV infection in macrophages. Interestingly, a similar glycolytic response was found in the few cells that survived HIV infection and silenced the virus as compared to uninfected cells (Fig. 3D). No changes in oligomycin response were detected in ECAR (Fig. 3E), supporting the conclusion that the metabolic effects of HIV are in the mitochondria and not on the glycolytic pathways. This is a significant difference from circulating viral reservoirs, which express increased amounts of GLUT-1 and show elevated glycolysis^39–44^. Thus, the metabolism of latently HIV infected macrophages is different from that in T cell viral reservoirs.

### Lipid droplets accumulate in surviving HIV infected macrophages and associate with enlarged mitochondria

**Figure 4:**
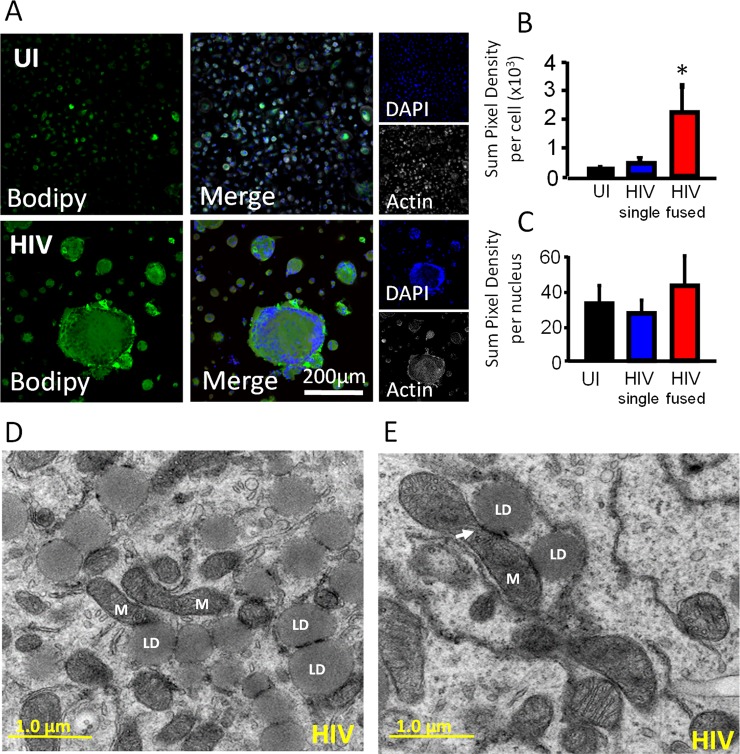
HIV infection increased lipid accumulation and interaction of lipids with the mitochondria. Cells were fixed with 4% PFA but not permeabilized to prevent lipid loss. Next, cells were stained with DAPI, BODIPY, and phalloidin to label nuclei, lipids, and actin, respectively. (**A**) A representative example of uninfected (UI) and HIV infected (HIV) macrophage cultures staining used to measure lipid production at the late stage of infection. (**B**) Quantification of lipid staining intensity indicates that HIV fused cells had a higher amount of lipids as compared to uninfected cells (UI) or HIV unfused cells (HIV single). *p = 0.0230, n = 6. (**C**) Quantification of lipids per nucleus was normalized to the number of nuclei per cell, with no significant difference per nucleus between uninfected cultures and HIV infected cultures. (**D**,**E**) Showing two different examples of proliferating and fused mitochondria (M) and their close interaction with lipid droplets (LD), see the arrow.

Lipid dysregulation is a well-documented problem in the HIV infected population, and it is mostly associated with foam macrophages (uninfected and HIV infected) that survive for extended periods of time, resulting in increased risk for cardiovascular disease, atherosclerosis, and dementia^45–48^. To determine whether the changes in mitochondrial fusion and metabolism are due to alterations in lipid metabolism, BODIPY staining was performed at different stages post infection. Our data indicate that HIV infection of human macrophages results in significant accumulation of lipids in the macrophages that survive HIV infection, especially in the fused macrophages (Fig. 4A). Quantification of lipid accumulation using confocal microscopy indicated that HIV fused cells accumulate more lipids than uninfected and single HIV infected cells (Fig. 4B, *p = 0.00230, n = 6). Lipid accumulation was not due to the cell to cell fusion due that calibration of the numbers of lipid bodies per nuclei was insignificant (Fig. 4C, HIV fused). However, examination of HIV-infected surviving cells using electron microscopy indicates that lipid distribution was altered. Under HIV conditions, mitochondria (M) become bigger (Fig. 1F) and closely associated with lipid droplets. Activation of human macrophages with TNF-α plus IFN-γ did not result in a similar metabolic profile (data not shown). Thus, these metabolic changes are again associated with the HIV infection and not due to general immune activation. These data indicate that HIV surviving cells accumulate lipids and direct lipid droplets to areas rich in enlarged mitochondria.

### Tricarboxylic acid metabolites generated prior to the mitochondrial steps are key metabolites in lipid accumulation and tricarboxylic acid (TCA) cycle dysregulation

**Figure 5:**
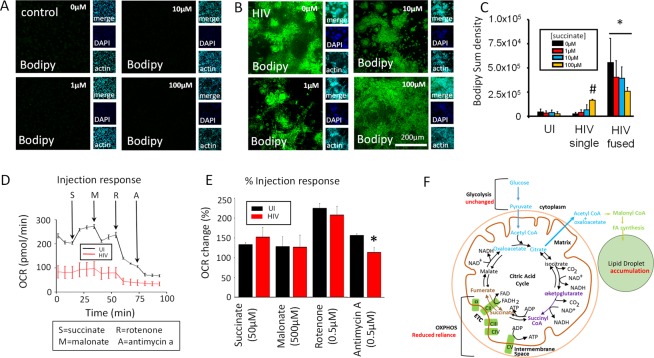
Succinate and α-KG are key TCA intermediates in lipid accumulation. (**A**) Representative examples of control and (**B**) HIV infected macrophages in untreated (0 µm) and treated with 1, 10, and 100 µM succinate and stained with BODIPY (lipid, green stain), DAPI (nuclei, blue stain), and actin (cyan stain). (**C**) Measurement of BODIPY staining per cell quantified using Nikon Elements software. Control (UI) cells did not respond to succinate treatment. Single cells in HIV infected cultures accumulated lipids in a dose dependent manner according to increased concentration of succinate (HIV single, ^#^p = 0.0002). Fused HIV infected macrophages accumulated lipids in response to succinate treatment. *p ≤ 0.0230, n = 4 different individuals. (**D**) A representative example of an OCR response curve in response to 50 µM succinate (S), 500 µM malonate (M), 0.5 µM rotenone, and 0.5 µM Antimycin-A (**A**). (**E**) Quantification of the OCR change in uninfected and HIV infected macrophages. Only changes in antimycin A response were found to be significant (*p = 0.0045, n = 7). (**F**) The cartoon represents a summary of the data and the proposed hypothesis of how lipids are accumulated in HIV infected macrophages. Red letters indicate our data. Purple letters indicate the affected areas supported by our data. Blue letters indicate the areas probably involved in lipid accumulation, but not tested in this study.

Our data so far indicate no changes in glycolysis, increased lipid accumulation, and reduced OXPHOS reliance, suggesting that the TCA cycle is compromised and may provide a stable alternative source of energy in addition to glucose, such as lipids. Furthermore, the increase in lipids indicates that there is an additional source of carbons to accumulate large lipid bodies. Therefore, we hypothesized that an additional source of carbon was contributing to the TCA cycle to fuel lipid accumulation and OXPHOS function. To test this idea, we treated cultures of human macrophages with different concentrations of succinate (1, 10 and 100 µM, a metabolite generated prior to the OXPHOS steps), and lipid accumulation was determined by confocal microscopy and OCR quantifications. Addition of succinate to uninfected cultures did not change lipid abundance at any concentration tested (control, Fig. 5A).

In contrast, succinate treatment of HIV infected cultures of macrophages increased lipid accumulation at all concentrations tested (Fig. 5B, representative images). Quantification of BODIPY staining confirmed that succinate treatment of uninfected cultures did not alter lipid loading (Fig. 5C, UI). Overall, HIV infection increased lipid accumulation (combination of HIV single cells + HIV fused cells, Fig. 5C), but most of the lipid loading was observed in HIV fused macrophages, which are the cells that survive infection and silence viral replication (Fig. 5C, *p = 0.0023 as compared to UI cells, n = 4).

Furthermore, OCR determinations in response to succinate, malonate, and rotenone demonstrated no differences between uninfected and HIV infected conditions, with the OCR recovering to control levels. The only difference observed was response to antimycin A, an inhibitor of complex IV, which demonstrates a difference in OXPHOS function associated with complex IV (Fig. 5E, *p = 0.0045, n = 7). Activation of human macrophages with TNF-α plus IFN-γ did not result in similar metabolic changes (data not shown), indicating these metabolic changes are associated with the HIV infection and not due to general immune activation. Together, these results show that HIV reservoirs have a compromised TCA cycle and OXPHOS system, with the accumulating lipids possibly serving as an additional source of energy to support the minimal loss in OCR function (see Fig. 5F).

### HIV reservoirs use glutamine/glutamate as an alternative fuel source to generate energy

**Figure 6:**
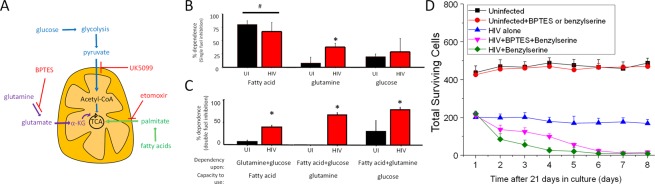
HIV infection regulates the metabolism of latently infected macrophages. (**A**) Diagram of mitochondrial fuel input from glycolytic, glutamine, and fatty acid pathways, with appropriate inhibitors used to prevent the usage of these pathways. (**B**) OCR changes used to measure percentage dependency of one or two fuel types. OCR changes are measured at baseline (no compounds) for roughly 17 minutes, followed by injection of one fuel inhibitor for single fuel dependency, or with two inhibitors for double fuel dependency. OCR changes resulting from treatment are measured for approximately 40 minutes, followed by injection of the remaining two fuels for single fuel dependency experiments, or the single remaining fuel for double dependency measurements. Dependency for single and double fuel experiments is calculated as the percentage change from baseline due to the first injection. (**B**) Mitochondrial OXPHOS dependency on fatty acid, glutamine, and glucose. HIV infected macrophages were more dependent on glutamine than their uninfected counterparts (*p = 0.0001, n = 4). There was no significant change in dependency on fatty acid or glucose for OXPHOS during HIV infection. There was a significant dependence on fatty acids for OXPHOS in uninfected and HIV infected cells compared to glutamine and glucose (^#^p ≤ 0.0119). (**C**) Analysis of mitochondrial dependence on two fuel types for OXPHOS. When forced to use a single fuel source for OXPHOS, there was a significant decrease in the ability for HIV macrophages to use fatty acids and glucose, and a significant increase in the ability to use glutamine (*p ≤ 0.0002). Single and double dependency experiments indicate that uninfected macrophages are flexible to shift among several types of energy sources. However, HIV infected macrophages have an increased “flexibility” to shift among these forms of energy. (**D**) To evaluate whether the use of glutamine, glutamate, and α-KG is necessary for the survival of HIV reservoirs, we treated uninfected and HIV infected cultures of surviving macrophages with BPTES or Benzylserine. Both inhibitors did not alter the survival of uninfected cultures. However, the combination of both or Benzylserine alone resulted in significant cell death in the HIV infected cells. All points are significant after two days post-treatment (n = 4, p ≤ 0.005).

Normally, cells use glucose and fatty acids as a major source of energy; however, in pathological conditions, such as neuro/glioblastoma and other types of cancer, amino acids such as glutamine are used as fuel sources, mainly to avoid a dependency on carbon sources from the bloodstream^49–51^. Critically, glutamine/glutamate can provide additional energy to the TCA cycle by providing extra alpha-ketoglutarate (α-KG) and succinate as shown in Fig. 6A. Thus, to determine whether glutamate/glutamine could provide an additional source of carbon to the TCA cycle, single fuel dependency was determined. We measured OXPHOS by inhibiting fatty acid contribution to the TCA with etomoxir (fatty acid oxidation inhibitor), UK5099 (glucose oxidation inhibitor), or BPTES (glutaminase inhibitor) as described in Fig. 6A. Addition of one inhibitor at a time (single fuel dependency, Fig. 6B) showed that uninfected macrophages mostly used fatty acids and glucose to produce ATP (UI, Fig. 6B). As expected, minimal to undetectable use of glutamine as a source of energy was found in uninfected conditions (Fig. 6B, UI, glutamine). In HIV infected macrophages, while fatty acids and glucose were still utilized, the contribution of glutamine sources significantly increased (Fig. 6B, HIV, glutamine, *p = 0.0001, n = 4). These data indicate that HIV infected macrophages that survive infection behave similarly to cancer cells treated with Bis-2-(5-phenylacetamido-1,3,4-thiadiazol-2-yl)ethyl sulfide, BPTES, an inhibitor of glutaminase^52^. Also, activation of human macrophages with TNF-α plus IFN-γ did not result in similar metabolic changes indicating that this phenotype is unique (data not shown). Thus metabolic changes are associated with the HIV infection and not due to general immune activation. Furthermore, the changes in glutamine/glutamate/α-KG dependency were associated with higher expression of glutaminase and glutamine synthetase (data not shown). Therefore, like cancer cells, HIV infected cells that survive infection use particular amino acid pathways as a significant source of energy.

### HIV reservoirs gain the flexibility to shift between different sources of energy

To assess the flexibility of uninfected and HIV infected cells to shift among different sources of energy, we determined the capacity of cells to use either fatty acids, glutamine, or glucose alone by blocking two major sources of energy at a time. If the cells were exclusively reliant on the two inhibited fuel sources, we expected to observe a drop in OCR. In contrast, if the cells were capable of using the remaining fuel source, then there should be little to no change in the OCR.

Fuel reliance on cells under HIV conditions was significantly altered compared to uninfected cells (Fig. 6C, *p ≤ 0.0002). If we simultaneously inhibited glucose and glutamine oxidation using a cocktail of etomoxir and UK5099, thus requiring cells to use fatty acids, the OCR change in uninfected cells was negligible (Fig. 6C, fatty acid, UI, black bar). These data indicate that uninfected macrophages can use fatty acids as a fuel source for OXPHOS, and is consistent with our observation that macrophages are dependent on fatty acids more than other fuel types (Fig. 6A, fatty acid compared to glutamine and glucose). However, HIV infected macrophages were even more capable of further using fatty acids as a fuel source when it was the only fuel source available (Fig. 6C, fatty acid, HIV, red bar).

When we simultaneously inhibited glucose and fatty acid oxidation using a cocktail of UK5099 and etomoxir, we observed that uninfected macrophages could not use glutamine for OXPHOS (Fig. 6C, glutamine, UI, black bar), indicating their strong dependence upon fatty acid and glucose for OXPHOS. In contrast, when HIV infected macrophages were assessed, they showed the ability to use glutamine as fuel for OXPHOS when the other sources were unavailable (Fig. 6C, glutamine, red bar). If fatty acid and glutamine dependency were blocked, uninfected and HIV infected cells were able to increase their reliance on glucose (Fig. 6C, glucose, red bar).

Based on the increased dependency on glutamine in surviving HIV infected macrophages, we hypothesized that survival of these cells was also dependent on glutamine, glutamate, and α-KG metabolism. To demonstrate that, we treated late stage surviving macrophages, uninfected and HIV infected, with BPTES, a glutaminase inhibitor (1 µM, BPTES), or benzylserine (0.3 mM), an ASCT2 inhibitor, and the numbers of total surviving cells were quantified using microscopy (Fig. 6D). Uninfected cultures maintained for 21 days and then subjected to the blockers did not result in any cell death (Fig. 6D, black and red lines). In contrast, the treatment with BPTES (Fig. 6D) plus benzylserine reduced the numbers of surviving cells (Fig. 6D). Indeed, results of treatment of surviving HIV infected cultures with Benzylserine alone were similar to use of the combination of both inhibitors (Fig. 6D).

In conclusion, our results indicate that glutamate, glutamine, and α-KG are essential metabolites to preserve the survival of HIV reservoirs. Also, activation of human macrophages with TNF-α plus IFN-γ did not result in similar metabolic changes (data not shown). Thus, these metabolic changes are associated with the HIV infection and not due to general immune activation. Together, these experiments showed that HIV infected cells acquired the ability to use glutamine as a major source of energy and to shift between different sources of energy.

## Discussion

Our recently published study showed that a small population of human macrophages survive acute HIV infection and that these surviving infected cells become latently infected, as viral replication is not detected^23^. In the present study, we show that the surviving cells exhibit metabolic compromise and mitochondrial fusion, lose reliance on OXPHOS, and accumulate lipids. Most of these changes could be mimicked by adding succinate or glutamine/glutamate to the cells, supporting a compromised TCA cycle. Despite these changes, no overall alterations in mitochondrial membrane potential or ETC mitochondrial expression were detected. We also showed that while uninfected macrophages exclusively use glucose and fatty acids as major sources of energy, latently HIV infected macrophages use glutamine/glutamate as a significant source and gain the capability to shift from one metabolic source to another. Furthermore, blocking the use of glutamate, glutamine, or α-KG results in the specific elimination of HIV surviving reservoirs. Together, these data identify a unique metabolic signature of latently infected cells (not replicated by immune activation), which could be pharmacologically targeted to eliminate HIV reservoirs.

Our original study, using several CNS cell types, including astrocytes, microglia, and macrophages, indicated that HIV infection, even in the absence of replication, deftly changes the entire metabolism of the cell. Based on that study, the current study, and other reports, we hypothesize that HIV effects specific mitochondrial changes in order to 1. Prevent apoptosis of the infected cell, allowing it to become a viral reservoir^23,26,28^; 2. Reduce their energetic dependency and metabolism to abolish immune recognition; 3. Use mitochondrial factors to amplify apoptosis and toxicity maintaining the survival of the few infected cells^23,24,53,54^; 4. Use viral proteins to maintain survival and HIV associated inflammation^55,56^; 5. Activate a “cancer-like program” to survive independently of efficient blood circulation^57,58^; 6. Activate novel forms of viral reactivation^8,24,59,60^; 7. Lastly, we propose that blocking these metabolic pathways could contribute to killing viral reservoirs. Our previous work in human macrophages indicates that HIV blocks the formation of the apoptosome and that in surviving cells Bim, a highly apoptotic protein, is recruited to the mitochondria without resulting in apoptosis^23^, suggesting that in HIV surviving cells, mitochondrial function is compromised. Therefore, HIV uses the mitochondria not only to prevent apoptosis but also to maintain the survival of the HIV infected cells.

There are numerous reports that HIV and ART, including NRTI (nucleoside reverse transcriptase inhibitors), low nadir CD4, aging, and high body mass are directly associated with HIV associated mitochondrial dysfunction^61–64^. However, this damage has been attributed to non-specific effects of the virus and ART. In contrast, we propose that the virus also controls mitochondrial function, because it provides specific advantages to the virus, including promoting the survival of latently infected macrophages, allowing them to become viral reservoirs.

Interestingly, the main attention on metabolism in the context of HIV has been focused on circulating cells and glucose transporter-1 (GLUT-1), which is upregulated in HIV infected individuals irrespective of ART treatment and is associated with immune activation^65,66^. These results imply that active glycolysis and OXPHOS are the main sources of energy in circulating HIV infected cells^40,66,67^. However, we observed that infected cells acquired the ability to use glutamate/glutamine as a major energy source (Fig. 6). Interestingly, several reports focusing on glioblastoma have suggested that the use of glutamate/glutamine provided cancer cells with several advantages over using glucose and lipids, such as being independent of blood circulation and oxygen tension^68–70^. Currently, only a few reports support a role of glutamine/glutamate as a major source of energy in HIV^71–73^. For instance, two recent independent reports indicated that in the periphery, CD4^+^ T cell counts were inversely correlated with high serum levels of glutamine and glucose, suggesting a role of these metabolites early on in infection and viral spread^71,74^. Therefore, glutamine becomes an essential metabolite in early stages of HIV infection, but also is essential during the generation and survival of viral reservoirs.

In macrophages, especially those from the brain, the dysregulation of glutamine/glutamate metabolism becomes more relevant because glutamate/glutamine levels are highly regulated by glial cells^75–77^. We have demonstrated that glial cells are dysregulated by HIV infection, even in the absence of viral replication, by amplifying neurotransmitter dysfunction via gap junction channels^27,29,54,56,78^. Furthermore, the use of this alternative fuel could provide an unlimited source of energy for HIV infected cells to survive within the brain. Several different neuroimaging experiments support this novel hypothesis: first, the numbers of infected cells are low^1,79,80^; second, changes in brain volume in the current ART era do not correlate with viral replication^81–84^; third, currently few neuronal markers of acute damage are found by magnetic resonance spectroscopy, MRS^53,85–87^; fourth, the main compromised metabolites are related to glutamine as found in our studies^85,88–92^. Glutamine can also be potentially used to produce nucleotides for DNA replication or signaling, including ATP and GMP^93^, both of which are essential for cell to cell signaling and viral replication. Therefore, local glutamate/glutamine dysregulation could be used as a biomarker of HIV infection, be exploited in potential treatments, and could also explain the increased susceptibility of infected individuals to several types of cancer that may also use glutamine/glutamate as a major source of energy to survive^22,94^. Our experiments blocking glutaminase and ASCT2 indicate a specific killing of surviving HIV infected macrophages, but not uninfected macrophages. ASCT2 is the major transporter responsible for glutamine uptake into the mitochondria and has been targeted in several cancers resulting in decreased growth and proliferation^95,96^. Thus, targeting these pathways is an attractive potential target to eliminate viral reservoirs.

Various potential biomarkers of HIV CNS disease have been proposed by several groups, including neopterin, neurofilament-light (NFL), BC11B, beta-2-microglobulin, several markers of inflammation (sCD163, CCL2, TNF-α, IL-6, sCD14, and CXCL10), and interferon-alpha^97–100^. However, all these biomarkers are associated with already occurring tissue damage and do not predict future damage. Only recently NIH sponsored groups such as CHARTER, NNTC, Neuroimaging Consortium, and proteomic determinations done by several laboratories have indicated that local alterations in metabolites could predict disease onset. Some of these metabolites and mitochondrial markers are citrate, creatinine, glutamine, glucose, inositol, glutamic acid, and CSF mtDNA^99,101–104^. Thus, HIV infection, even in the absence of replication, has profound effects on the metabolism of infected cells, which may help to perpetuate the virus or promote the survival of infected cells.

In agreement with our data, several viruses, such as HCV, Kaposi sarcoma herpesvirus (KSHV), adenovirus, and dengue virus, use glutamine-derived carbons as a significant source of energy during their pathogenesis^105–109^. As described above, the best-described disease where glutamine is used for energy is cancer, especially neuroblastoma^110–112^. Studies of glioblastoma demonstrate that glutamine is not only a substrate for several enzymes involved in neurotransmission and metabolism but is also essential for the synthesis of nucleotides, other amino acids, and glucosamine^113,114^, all of which are essential for HIV infection, the formation of viral reservoirs, and reactivation. Several types of cancers, including glioblastoma, exhibit a high dependency on glutamine, also called “glutamine addiction,” and inhibition of these pathways has been shown to reduce tumor growth and induce cancer cell death^115–118^. We suggest that this avenue should also be explored as an alternative treatment to reduce or eliminate viral reservoirs.

## Experimental Procedures

### Reagents

Unless otherwise indicated, all reagents were purchased from Sigma (St. Louis, MO). HIV_ADA_, CEM cells, and antiretrovirals were from the NIH AIDS Research and Reference Reagent Program (Germantown, MD). Medium, penicillin/streptomycin (P/S), dyes and secondary antibodies were obtained from Thermo-Fisher (Waltham, MA). Human AB serum and FBS were from Lonza (Walkersville, MD). HEPES was from USB (Cleveland, OH). HIV-p24 ELISA was obtained from Perkin-Elmer (Waltham, MA). Antibodies to HIV-p24 were obtained from Genetex (Irvine, CA). All other antibodies were purchased from Sigma, Santa Cruz (Santa Cruz, CA) or Abcam (Cambridge, MA). Purified mouse IgG_2B_ and IgG_1_ myeloma protein were from Cappel Pharmaceuticals, Inc. TUNEL was obtained from Roche Ltd (Germany). All experiments were performed under the regulations of Rutgers University and the NIH.

### Monocyte isolation and macrophage culture

Human monocytes were isolated from leukopaks obtained from the New York Blood Center. Peripheral blood mononuclear cells (PBMCs) were isolated by differential centrifugation using a Ficoll gradient (GE Healthcare, Piscataway, NJ). Adherent cells were cultured for seven days in the presence of 10 ng/ml macrophage colony stimulating factor (Miltenyl Biotec, San Diego, CA) in RPMI 1640 with 10% FBS, 5% human AB serum, 1% P/S, and 10 mM HEPES to differentiate the cells into macrophages.

### HIV infection and replication

After seven days in culture to enable differentiation, macrophages were inoculated with 20–50 ng/ml HIV_ADA_ for 24 hours, and then apoptosis, fusion, and expression of apoptotic proteins were examined. Supernatants were collected, and the medium was changed every 24 hours until 7, 14, 21 and 28 days post-inoculation. Viral replication was analyzed by HIV p24 ELISA according to the manufacturer’s instructions. Also, to assure that our cells were infected, we performed HIV DNA staining and Alu-PCR to detect infection as we recently described^23^. In all our cultures, HIV infection was detected (data not shown).

### Western blot

Protein levels were determined by immunoblot as previously described^119,120^. Briefly, lysate or immunoprecipitated samples were sonicated for 10 s (Microson ultrasonic cell disrupter, Heat systems). Protein content was determined by the Bradford method obtained from Bio-Rad. Aliquots of proteins in denaturing buffer were resolved in 10% SDS-PAGE and then electrotransferred to nitrocellulose. Non-specific protein binding was blocked with 5% non-fat milk in Tris-buffer for 30 min, followed by overnight incubation with primary antibodies obtained from Abcam (mitochondrial complexes I-V and loading control VDAC). The membrane was washed with TBS and then incubated with anti-mouse, anti-goat or anti-rabbit IgG antibody conjugated to alkaline phosphatase. Antigen-antibody complexes were detected with ECL reagent.

### Immunofluorescence

Human macrophages, HIV-infected and uninfected, were grown on glass coverslips, fixed and permeabilized in 70% ethanol for 20 min at −20 °C or fixed in 4% paraformaldehyde and permeabilized with 0.01% Triton-X for 2 minutes. Cells were incubated in TUNEL reaction mixture (Roche, Germany) at 37 °C for one h, washed three times with PBS and incubated in blocking solution for 30 min at room temperature. Cells were incubated in blocking solution for 30 min at room temperature and then in primary antibody (anti-HIV-p24, TOM-20, mitotracker, BODIPY or isotype controls) overnight at 4 °C. Cells were washed several times with PBS at room temperature and incubated with the proper secondary labeled antibody (Thermo-Fisher, Carlsbad, CA) for one h at room temperature, followed by another wash in PBS for one h. Then, cells were mounted using anti-fade reagent with DAPI. Cells were examined by confocal microscopy using an A_1_ Nikon (Tokyo, Japan) to quantify the total numbers of cells as well as TUNEL positive cells.

### Electron microscopy

Fresh samples were fixed with 2.5% glutaraldehyde, 2% paraformaldehyde in 0.1 M sodium cacodylate buffer, postfixed in 1% osmium tetroxide followed by 2% uranyl acetate, dehydrated through a graded series of ethanol and embedded in LX112 resin (LADD Research Industries, Burlington, VT). Ultrathin sections were cut on a Reichert Ultracut UCT, stained with uranyl acetate followed by lead citrate and viewed on a JEOL 1200EX transmission electron microscope at 80 kV.

### SeaHorse-extracellular flux analyzer

Cell Respiratory Assay OCR and ECAR were measured using the XFp extracellular flux analyzer (Seahorse Biosciences, now Agilent Technologies) as described by the manufacturer.

### Statistical analysis

Data were analyzed using Origin 8.1 (Northampton, MA, US). For single comparisons, Student’s t-test was performed. For multiple comparisons, mean differences were tested by non-parametric Kruskal–Wallis analysis and adjusted by use of the Bonferroni–Dunn correction. p values of <0.05 were considered significant.

## Supplementary information

Supplemental

## References

[1] Wong JK, Yukl SA (2016). Tissue reservoirs of HIV. Curr Opin HIV AIDS.

[2] Kimata JT, Rice AP, Wang J (2016). Challenges and strategies for the eradication of the HIV reservoir. Curr Opin Immunol.

[3] Cary DC, Fujinaga K, Peterlin BM (2016). Molecular mechanisms of HIV latency. J Clin Invest.

[4] Siliciano RF, Greene WC (2011). HIV latency. Cold Spring Harb Perspect Med.

[5] Gordon S, Taylor PR (2005). Monocyte and macrophage heterogeneity. Nat Rev Immunol.

[6] Philpott SM (2003). HIV-1 coreceptor usage, transmission, and disease progression. Curr HIV Res.

[7] Groot F, Welsch S, Sattentau QJ (2008). Efficient HIV-1 transmission from macrophages to T cells across transient virological synapses. Blood.

[8] Eugenin, E. A., Gaskill, P. J. & Berman, J. W. Tunneling nanotubes (TNT) are induced by HIV-infection of macrophages: a potential mechanism for intercellular HIV trafficking. *Cell Immunol* **254**, 142–148, S0008-8749(08)00157-3 (2009). 10.1016/j.cellimm.2008.08.005 PMC2701345 18835599

[9] Eugenin EA, Gaskill PJ, Berman JW (2009). Tunneling nanotubes (TNT): A potential mechanism for intercellular HIV trafficking. Communicative & integrative biology.

[10] Honeycutt JB (2016). Macrophages sustain HIV replication *in vivo* independently of T cells. J Clin Invest.

[11] Raposo G (2002). Human macrophages accumulate HIV-1 particles in MHC II compartments. Traffic.

[12] Pelchen-Matthews A, Kramer B, Marsh M (2003). Infectious HIV-1 assembles in late endosomes in primary macrophages. J Cell Biol.

[13] Arainga M (2017). A mature macrophage is a principal HIV-1 cellular reservoir in humanized mice after treatment with long acting antiretroviral therapy. Retrovirology.

[14] Bellingan GJ, Caldwell H, Howie SE, Dransfield I, Haslett C (1996). *In vivo* fate of the inflammatory macrophage during the resolution of inflammation: inflammatory macrophages do not die locally, but emigrate to the draining lymph nodes. J Immunol.

[15] Murphy J, Summer R, Wilson AA, Kotton DN, Fine A (2008). The prolonged life-span of alveolar macrophages. Am J Respir Cell Mol Biol.

[16] Lassmann H, Hickey WF (1993). Radiation bone marrow chimeras as a tool to study microglia turnover in normal brain and inflammation. Clin Neuropathol.

[17] Melnicoff MJ, Horan PK, Breslin EW, Morahan PS (1988). Maintenance of peritoneal macrophages in the steady state. J Leukoc Biol.

[18] Dumas F, Haanappel E (2017). Lipids in infectious diseases - The case of AIDS and tuberculosis. Biochim Biophys Acta.

[19] Funderburg NT, Mehta NNL (2016). Abnormalities and Inflammation in HIV Inflection. Curr HIV/AIDS Rep.

[20] Pereira MSL, Klamt F, Thome CC, Worm PV, de Oliveira DL (2017). Metabotropic glutamate receptors as a new therapeutic target for malignant gliomas. Oncotarget.

[21] Luo X (2017). Emerging roles of lipid metabolism in cancer metastasis. Mol Cancer.

[22] Tardito S (2015). Glutamine synthetase activity fuels nucleotide biosynthesis and supports growth of glutamine-restricted glioblastoma. Nat Cell Biol.

[23] Castellano P, Prevedel L, Eugenin EA (2017). HIV-infected macrophages and microglia that survive acute infection become viral reservoirs by a mechanism involving Bim. Sci Rep.

[24] Okafo G, Prevedel L, Eugenin E (2017). Tunneling nanotubes (TNT) mediate long-range gap junctional communication: Implications for HIV cell to cell spread. Sci Rep.

[25] Malik S, Eugenin EA (2016). Mechanisms of HIV Neuropathogenesis: Role of Cellular Communication Systems. Curr HIV Res.

[26] Orellana JA (2014). HIV increases the release of dickkopf-1 protein from human astrocytes by a Cx43 hemichannel-dependent mechanism. J Neurochem.

[27] Eugenin EA, Berman JW (2013). Cytochrome C dysregulation induced by HIV infection of astrocytes results in bystander apoptosis of uninfected astrocytes by an IP3 and calcium-dependent mechanism. J Neurochem.

[28] Eugenin EA, Clements JE, Zink MC, Berman JW (2011). Human immunodeficiency virus infection of human astrocytes disrupts blood-brain barrier integrity by a gap junction-dependent mechanism. J Neurosci.

[29] Eugenin EA, Berman JW (2007). Gap junctions mediate human immunodeficiency virus-bystander killing in astrocytes. J Neurosci.

[30] Honeycutt, J. B. *et al*. HIV persistence in tissue macrophages of humanized myeloid-only mice during antiretroviral therapy. *Nat Med*, 10.1038/nm.4319 (2017). 10.1038/nm.4319 PMC5419854 28414330

[31] Graziano F, Vicenzi E, Poli G (2016). Immuno-Pharmacological Targeting of Virus-Containing Compartments in HIV-1-Infected Macrophages. Trends Microbiol.

[32] Prevedel, L. *et al*. Identification, Localization, and Quantification of HIV Reservoirs Using Microscopy. *Curr Protoc Cell Biol*, e64, 10.1002/cpcb.64 (2018). 10.1002/cpcb.64 PMC6386609 30265439

[33] Morita M (2017). mTOR Controls Mitochondrial Dynamics and Cell Survival via MTFP1. Mol Cell.

[34] Rambold AS, Cohen S, Lippincott-Schwartz J (2015). Fatty acid trafficking in starved cells: regulation by lipid droplet lipolysis, autophagy, and mitochondrial fusion dynamics. Dev Cell.

[35] Gomes LC, Di Benedetto G, Scorrano L (2011). During autophagy mitochondria elongate, are spared from degradation and sustain cell viability. Nat Cell Biol.

[36] Pfleger J, He M, Abdellatif M (2015). Mitochondrial complex II is a source of the reserve respiratory capacity that is regulated by metabolic sensors and promotes cell survival. Cell Death Dis.

[37] Wheaton WW (2014). Metformin inhibits mitochondrial complex I of cancer cells to reduce tumorigenesis. Elife.

[38] Tormos KV (2011). Mitochondrial complex III ROS regulate adipocyte differentiation. Cell Metab.

[39] Palmer CS (2017). Metabolically active CD4+ T cells expressing Glut1 and OX40 preferentially harbor HIV during *in vitro* infection. FEBS Lett.

[40] Masson JJR (2017). Assessment of metabolic and mitochondrial dynamics in CD4+ and CD8+ T cells in virologically suppressed HIV-positive individuals on combination antiretroviral therapy. PLoS One.

[41] Palmer CS, Cherry CL, Sada-Ovalle I, Singh A, Crowe SM (2016). Glucose Metabolism in T Cells and Monocytes: New Perspectives in HIV Pathogenesis. EBioMedicine.

[42] Palmer CS (2014). Glucose transporter 1-expressing proinflammatory monocytes are elevated in combination antiretroviral therapy-treated and untreated HIV + subjects. J Immunol.

[43] Craveiro M, Clerc I, Sitbon M, Taylor N (2013). Metabolic pathways as regulators of HIV infection. Curr Opin HIV AIDS.

[44] Loisel-Meyer S (2012). Glut1-mediated glucose transport regulates HIV infection. Proc Natl Acad Sci USA.

[45] Tudorache IF, Trusca VG, Gafencu AV (2017). Apolipoprotein E - A Multifunctional Protein with Implications in Various Pathologies as a Result of Its Structural Features. Comput Struct Biotechnol J.

[46] Crowe SM (2010). The macrophage: the intersection between HIV infection and atherosclerosis. J Leukoc Biol.

[47] Carr A (2008). Pathogenesis of cardiovascular disease in HIV infection. Curr Opin HIV AIDS.

[48] Mattson MP, Haughey NJ, Nath A (2005). Cell death in HIV dementia. Cell Death Differ.

[49] Fan J (2013). Glutamine-driven oxidative phosphorylation is a major ATP source in transformed mammalian cells in both normoxia and hypoxia. Mol Syst Biol.

[50] Wise DR (2008). Myc regulates a transcriptional program that stimulates mitochondrial glutaminolysis and leads to glutamine addiction. Proc Natl Acad Sci USA.

[51] Perez-Escuredo J (2016). Lactate promotes glutamine uptake and metabolism in oxidative cancer cells. Cell Cycle.

[52] Elgogary A (2016). Combination therapy with BPTES nanoparticles and metformin targets the metabolic heterogeneity of pancreatic cancer. Proc Natl Acad Sci USA.

[53] Valdebenito S, Barreto A, Eugenin EA (2018). The role of connexin and pannexin containing channels in the innate and acquired immune response. Biochim Biophys Acta.

[54] Malik S, Theis M, Eugenin EA (2017). Connexin43 Containing Gap Junction Channels Facilitate HIV Bystander Toxicity: Implications in NeuroHIV. Front Mol Neurosci.

[55] Prevedel L, Morocho C, Bennett MVL, Eugenin EA (2017). HIV-Associated Cardiovascular Disease: Role of Connexin 43. Am J Pathol.

[56] Berman JW (2016). HIV-tat alters Connexin43 expression and trafficking in human astrocytes: role in NeuroAIDS. J Neuroinflammation.

[57] Engin AB, Engin ED, Golokhvast K, Spandidos DA, Tsatsakis AM (2017). Glutamatemediated effects of caffeine and interferongamma on mercury-induced toxicity. Int J Mol Med.

[58] Soh H, Wasa M, Wang HS, Fukuzawa M (2005). Glutamine regulates amino acid transport and glutathione levels in a human neuroblastoma cell line. Pediatr Surg Int.

[59] Ariazi J (2017). Tunneling Nanotubes and Gap Junctions-Their Role in Long-Range Intercellular Communication during Development, Health, and Disease Conditions. Front Mol Neurosci.

[60] Eugenin EA, Gaskill PJ, Berman JW (2009). Tunneling nanotubes (TNT): A potential mechanism for intercellular HIV trafficking. Commun Integr Biol.

[61] McComsey GA (2008). Mitochondrial RNA and DNA alterations in HIV lipoatrophy are linked to antiretroviral therapy and not to HIV infection. Antivir Ther.

[62] Smith RL (2017). Beyond the polymerase-gamma theory: Production of ROS as a mode of NRTI-induced mitochondrial toxicity. PLoS One.

[63] Zhang Y (2014). Long-term exposure of mice to nucleoside analogues disrupts mitochondrial DNA maintenance in cortical neurons. PLoS One.

[64] Margolis AM, Heverling H, Pham PA, Stolbach A (2014). A review of the toxicity of HIV medications. J Med Toxicol.

[65] Palmer CS (2016). Regulators of Glucose Metabolism in CD4(+) and CD8(+) T Cells. Int Rev Immunol.

[66] Palmer CS (2014). Increased glucose metabolic activity is associated with CD4+ T-cell activation and depletion during chronic HIV infection. AIDS.

[67] Palmer CS (2016). Emerging Role and Characterization of Immunometabolism: Relevance to HIV Pathogenesis, Serious Non-AIDS Events, and a Cure. J Immunol.

[68] El Ansari R (2018). Altered glutamine metabolism in breast cancer; subtype dependencies and alternative adaptations. Histopathology.

[69] Yang L, Venneti S, Nagrath D (2017). Glutaminolysis: A Hallmark of Cancer Metabolism. Annu Rev Biomed Eng.

[70] Marquez J (2017). Glutamine Addiction In Gliomas. Neurochem Res.

[71] Hegedus A (2017). Evidence for Altered Glutamine Metabolism in Human Immunodeficiency Virus Type 1 Infected Primary Human CD4(+) T Cells. AIDS Res Hum Retroviruses.

[72] Datta PK (2016). Glutamate metabolism in HIV-1 infected macrophages: Role of HIV-1 Vpr. Cell Cycle.

[73] Huang Y (2011). Glutaminase dysregulation in HIV-1-infected human microglia mediates neurotoxicity: relevant to HIV-1-associated neurocognitive disorders. J Neurosci.

[74] Ziegler TR, Judd SE, Ruff JH, McComsey GA, Eckard AR (2017). Amino Acid Concentrations in HIV-Infected Youth Compared to Healthy Controls and Associations with CD4 Counts and Inflammation. AIDS Res Hum Retroviruses.

[75] Dabrowska, K. *et al*. Roles of glutamate and glutamine transport in ammonia neurotoxicity: state of the art and question marks. *Endocr Metab Immune Disord Drug Targets*, 10.2174/1871520618666171219124427 (2017). 10.2174/1871520618666171219124427 29256360

[76] Rimmele TS, Rosenberg PA (2016). GLT-1: The elusive presynaptic glutamate transporter. Neurochem Int.

[77] Sery O, Sultana N, Kashem MA, Pow DV, Balcar VJ (2015). GLAST But Not Least–Distribution, Function, Genetics and Epigenetics of L-Glutamate Transport in Brain–Focus on GLAST/EAAT1. Neurochem Res.

[78] Castellano P, Eugenin EA (2014). Regulation of gap junction channels by infectious agents and inflammation in the CNS. Front Cell Neurosci.

[79] Parikh UM, McCormick K, van Zyl G, Mellors JW (2017). Future technologies for monitoring HIV drug resistance and cure. Curr Opin HIV AIDS.

[80] Stein J, Storcksdieck Genannt Bonsmann M, Streeck H (2016). Barriers to HIV Cure. HLA.

[81] Duan L (2016). Structural and functional characterization of EIAV gp45 fusion peptide proximal region and asparagine-rich layer. Virology.

[82] Sanford, R., Fellows, L. K., Ances, B. M. & Collins, D. L. Association of Brain Structure Changes and Cognitive Function With Combination Antiretroviral Therapy in HIV-Positive Individuals. *JAMA Neurol*, 10.1001/jamaneurol.2017.3036 (2017). 10.1001/jamaneurol.2017.3036 PMC5833491 29131878

[83] Guha A (2016). Topographies of Cortical and Subcortical Volume Loss in HIV and Aging in the cART Era. J Acquir Immune Defic Syndr.

[84] Ances BM, Hammoud DA (2014). Neuroimaging of HIV-associated neurocognitive disorders (HAND). Curr Opin HIV AIDS.

[85] Holmes MJ (2017). Longitudinal increases of brain metabolite levels in 5-10 year old children. PLoS One.

[86] Winston A (2015). Differences in the direction of change of cerebral function parameters are evident over three years in HIV-infected individuals electively commencing initial cART. PLoS One.

[87] Zahr NM, Mayer D, Rohlfing T, Sullivan EV, Pfefferbaum A (2014). Imaging neuroinflammation? A perspective from MR spectroscopy. Brain Pathol.

[88] Schuettfort G (2014). Proton 1H- and Phosphorus 31P-MR spectroscopy (MRS) in asymptomatic HIV-positive patients. J Int AIDS Soc.

[89] Bairwa D (2016). Case control study: magnetic resonance spectroscopy of brain in HIV infected patients. BMC Neurol.

[90] Harezlak J (2014). Predictors of CNS injury as measured by proton magnetic resonance spectroscopy in the setting of chronic HIV infection and CART. J Neurovirol.

[91] Hua X (2013). Disrupted cerebral metabolite levels and lower nadir CD4+ counts are linked to brain volume deficits in 210 HIV-infected patients on stable treatment. Neuroimage Clin.

[92] Cohen RA (2010). Cerebral metabolite abnormalities in human immunodeficiency virus are associated with cortical and subcortical volumes. J Neurovirol.

[93] Wise DR, Thompson CB (2010). Glutamine addiction: a new therapeutic target in cancer. Trends Biochem Sci.

[94] Sontheimer H (2008). A role for glutamate in growth and invasion of primary brain tumors. J Neurochem.

[95] Chen L, Cui H (2015). Targeting Glutamine Induces Apoptosis: A Cancer Therapy Approach. Int J Mol Sci.

[96] van Geldermalsen M (2016). ASCT2/SLC1A5 controls glutamine uptake and tumour growth in triple-negative basal-like breast cancer. Oncogene.

[97] Van Zoest, R. A. *et al*. Structural brain abnormalities in successfully treated HIV infection: associations with disease and cerebrospinal fluid biomarkers. *J Infect Dis*, 10.1093/infdis/jix553 (2017). 10.1093/infdis/jix553 29069436

[98] Andreoni, M. *et al*. Biomarkers of monitoring and functional reserve of physiological systems over time in HIV: expert opinions for effective secondary prevention. *New Microbiol* **40** (2017). 28994444

[99] Mehta SR (2017). Cerebrospinal fluid cell-free mitochondrial DNA is associated with HIV replication, iron transport, and mild HIV-associated neurocognitive impairment. J Neuroinflammation.

[100] Hellmuth J (2016). Neurologic signs and symptoms frequently manifest in acute HIV infection. Neurology.

[101] Sailasuta N (2016). Neuronal-Glia Markers by Magnetic Resonance Spectroscopy in HIV Before and After Combination Antiretroviral Therapy. J Acquir Immune Defic Syndr.

[102] Wright PW (2015). Cerebral white matter integrity during primary HIV infection. AIDS.

[103] Drewes JL (2015). Quinolinic acid/tryptophan ratios predict neurological disease in SIV-infected macaques and remain elevated in the brain under cART. J Neurovirol.

[104] Dickens AM (2015). Cerebrospinal fluid metabolomics implicate bioenergetic adaptation as a neural mechanism regulating shifts in cognitive states of HIV-infected patients. AIDS.

[105] Levy PL (2017). Hepatitis C virus infection triggers a tumor-like glutamine metabolism. Hepatology.

[106] Thai M (2015). MYC-induced reprogramming of glutamine catabolism supports optimal virus replication. Nat Commun.

[107] Sanchez EL, Carroll PA, Thalhofer AB, Lagunoff M (2015). Latent KSHV Infected Endothelial Cells Are Glutamine Addicted and Require Glutaminolysis for Survival. PLoS Pathog.

[108] Chambers JW, Maguire TG, Alwine JC (2010). Glutamine metabolism is essential for human cytomegalovirus infection. J Virol.

[109] El-Bacha T (2016). 1H Nuclear Magnetic Resonance Metabolomics of Plasma Unveils Liver Dysfunction in Dengue Patients. J Virol.

[110] Zhang J, Pavlova NN, Thompson CB (2017). Cancer cell metabolism: the essential role of the nonessential amino acid, glutamine. Embo J.

[111] Venneti S, Thompson CB (2017). Metabolic Reprogramming in Brain Tumors. Annu Rev Pathol.

[112] Venneti S (2015). Glutamine-based PET imaging facilitates enhanced metabolic evaluation of gliomas *in vivo*. Sci Transl Med.

[113] Durand P, Golinelli-Pimpaneau B, Mouilleron S, Badet B, Badet-Denisot MA (2008). Highlights of glucosamine-6P synthase catalysis. Arch Biochem Biophys.

[114] Tanaka K (2015). Compensatory glutamine metabolism promotes glioblastoma resistance to mTOR inhibitor treatment. J Clin Invest.

[115] Bolzoni M (2016). Dependence on glutamine uptake and glutamine addiction characterize myeloma cells: a new attractive target. Blood.

[116] Ratnikov B (2015). Glutamate and asparagine cataplerosis underlie glutamine addiction in melanoma. Oncotarget.

[117] Fogal V (2015). Mitochondrial p32 is upregulated in Myc expressing brain cancers and mediates glutamine addiction. Oncotarget.

[118] Hassanein M (2013). SLC1A5 mediates glutamine transport required for lung cancer cell growth and survival. Clin Cancer Res.

[119] Eugenin EA, Branes MC, Berman JW, Saez JC (2003). TNF-alpha plus IFN-gamma induce connexin43 expression and formation of gap junctions between human monocytes/macrophages that enhance physiological responses. J Immunol.

[120] Eugenin EA, D’Aversa TG, Lopez L, Calderon TM, Berman JW (2003). MCP-1 (CCL2) protects human neurons and astrocytes from NMDA or HIV-tat-induced apoptosis. J Neurochem.

