# Supplementary material for: HIV infection and latency induce a unique metabolic signature in human macrophages
Source: Sci Rep. 2019 Mar 8;9:3941. doi: 10.1038/s41598-019-39898-5 (PMC6408492; doi:10.1038/s41598-019-39898-5)
Supplement: Supplementary file 1 — Supplemental [file 41598_2019_39898_MOESM1_ESM.docx]

**HIV infection and latency induce a unique metabolic signature in human macrophages**

Castellano Paul^1^, Prevedel Lisa. ^1^, Valdebenito Silvana^1^*^,2^,* and Eugenin Eliseo A.^1,2, *^

**Affiliations:** ^1^Public Health Research Institute (PHRI), Newark, NJ, USA.^2^Department of Neuroscience, Cell Biology and Anatomy, University of Texas Medical Branch (UTMB), Galveston, Texas, USA.

**Supplemental Figures**

**Supplemental Figure 1: HIV infected macrophages are protected from apoptosis in an independent manner of changes in Bcl-2, Mcl-1, and Hsp-70 and 27, but depended in increased Bim expression ^23^.** Macrophages were isolated and plated for the whole cell lysate collection for immunoblot. Cells were infected with 50 ng/mL HIV_ADA_, and the lysate was collected at 7, 14, and 21 days post infection**. (A)** The whole cell lysate was probed for pro-apoptotic and anti-apoptotic proteins that participate in MOM pore opening including Bcl-2, Mcl-1, Hsp-70 and -27. There was no significant change in the expression of all these proteins (n = 3). Quantification of bcl-2 (B), mcl-1 (C), hsp-70 (D) and hsp-27 (E) expression in uninfected and HIV-infected macrophages.

**Supplemental Figure 2: Different stages of HIV infection, cell death, and replication.** Representative confocal images of the corresponding stages of HIV replication and survival. DAPI (blue) staining was used to identify nuclei, phalloidin (green) was used to stain actin and outline cells, and antibodies are targeting HIV-p24 (cyan) were used to identify infected cells. No HIV replication was detected, or unspecific staining was detected at any time point (Control). During the early stages of HIV infection (1-3 days post-infection) minimal HIV-p24 staining as well as cell death (see Fig. 1) was detected. During the mid-stage of HIV-infection (3-14 day), HIV-p24 production was evident in most cells, and cell death was high (see Fig. 1). During the late stage of HIV-infection (10-21 days, bottom row of panels) the few fused macrophages remain in the culture, but minimal to undetectable cell death and replication were detected (see Fig. 1).

**Supplemental Figure 3: HIV infected macrophages with large mitochondria lack changes in mfn-1 and -2 associated with mitochondrial fusion.** Macrophages were isolated and plated for the whole cell lysate collection for immunoblot. Cells were infected with 50 ng/mL HIV_ADA_, and the lysate was collected at 7, 14, and 21 days post infection. (**A**) The whole cell lysate was probed for mfn-1 and -2, both proteins associated with mitochondrial fusion as denoted in the cartoon (B). There was no significant change in the expression of these proteins (n = 3). Quantification of mfn1 (c) and mfn2 (D) in uninfected and HIV-infected macrophages.

**Supplemental Figure 4: HIV infection of macrophages results in a mitochondrial loss.** Macrophages were plated for fluorescent microscopy using the same methods as in previous figures and fixed at 7, 14, and 21dpi. One hour before fixation, cells were incubated with 250 nM Mitotracker CMTMRos to measure mitochondrial membrane potential and fixed using 4% paraformaldehyde for 20 minutes. Using 4% PFA helps to retain Mitotracker fluorescence according to the manufacturer’s protocol. Prior to staining with TOM20, cells were permeabilized with pure methanol chilled at 4^o^C for 20 minutes, washed with PBS, and placed in blocking solution. Antibody staining was conducted using the same methodology as described for previous figures. (A) A representative example of control (UI) and HIV infected (HIV) macrophages used for quantification of fluorescent signal, stained for Mitotracker mitochondrial membrane potential-sensitive dyes (red stain), mitochondrial outer membrane marker TOM20 (green stain), DAPI (blue, nuclei stain), and actin (gray stain). (B) Quantification of Tom 20 means pixel intensity per cell. There were no significant changes between control and HIV infected macrophages in TOM20 mean pixel intensity per cell. The HIV population of cells was also separated into fused cells and single cells to determine if the different populations were significantly different from one another. No differences between fused cells or non-multinucleated cells were apparent in TOM20 signal. (n=6) (C) Quantification of Mitotracker means pixel intensity per cell. There were no significant changes between control and HIV infected macrophages in Mitotracker mean pixel intensity per cell. The HIV population of cells was also separated into fused cells and non-fused cells to determine if the different populations were significantly different from one another. No differences between multinucleated cells or non-fused cells were apparent in the mitotracker signal. (n=6). (D and E) Mitotracker and TOM20 mean pixel intensity signals normalized to nuclei number per cell. There was a significant decrease in Mitotracker and TOM20 signals in fused cells when normalized to the number of nuclei in multinucleated cells (*p = 0.0003, n=6). (F) TOM20: Mitotracker ratio to measure changes in mitochondrial membrane potential. There was no change in TOM20: Mitotracker ratio in control, HIV infected, multinucleated cells or non-multinucleated cells in HIV infected cultures (n=6).

**Supplemental Figure 5: HIV infection did not alter the expression of the ET complexes.** (A) Uninfected and HIV infected macrophage mRNA was collected (n = 7) and subjected to qRT-PCR for complexes I to IV. There was no significant difference between control (set up as 1) and HIV infected macrophages in the transcription of ETC complexes I-IV. β-globulin was used as a relative control. (B) A representative example of Western blots for the mitochondrial isolate from late stages of infection probed for ETC complexes I-V and VDAC used as a loading control (n = 5). Each graph represents the summary of 5 independent experiments. (C) Quantification of pixel intensity with mitochondrial voltage-dependent anion channel (VDAC). There were no significant changes in expression of ETC complexes I-V between control and HIV infected lysate.

**
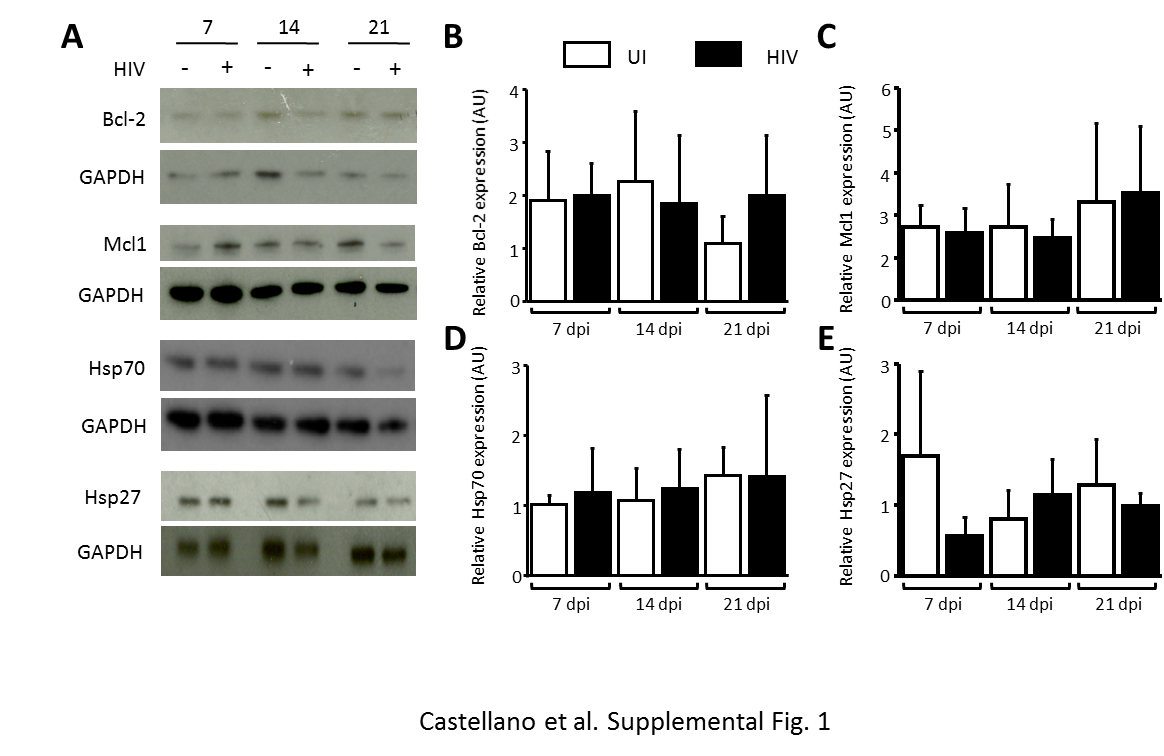
**

**
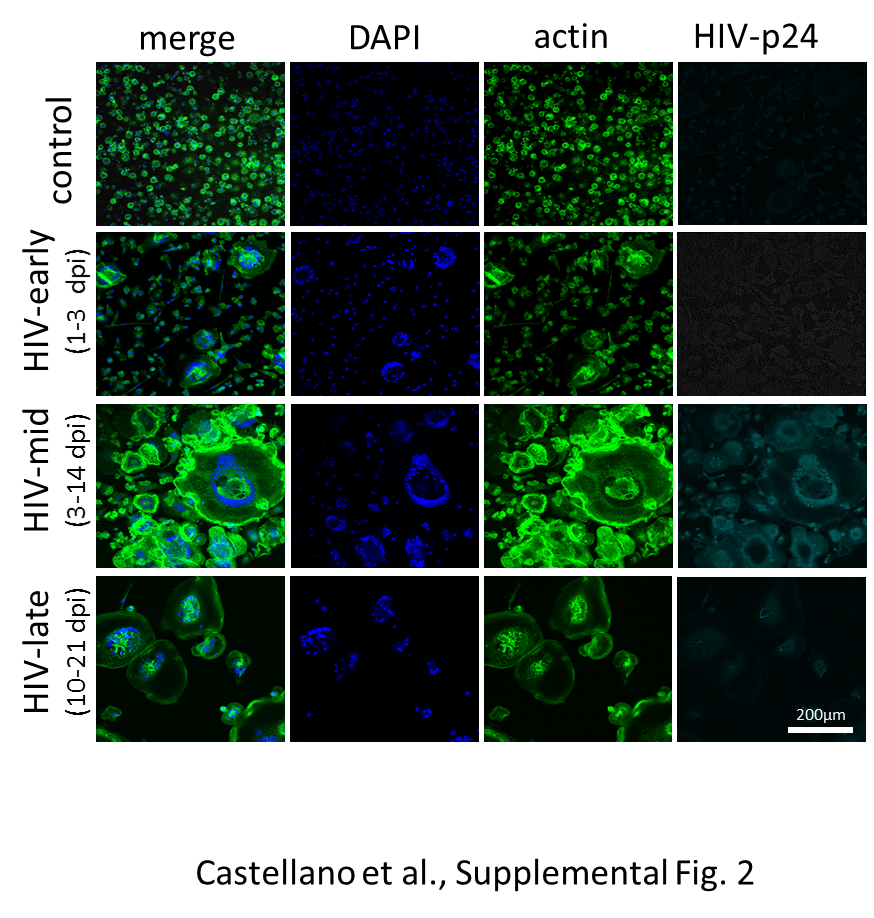
**

**
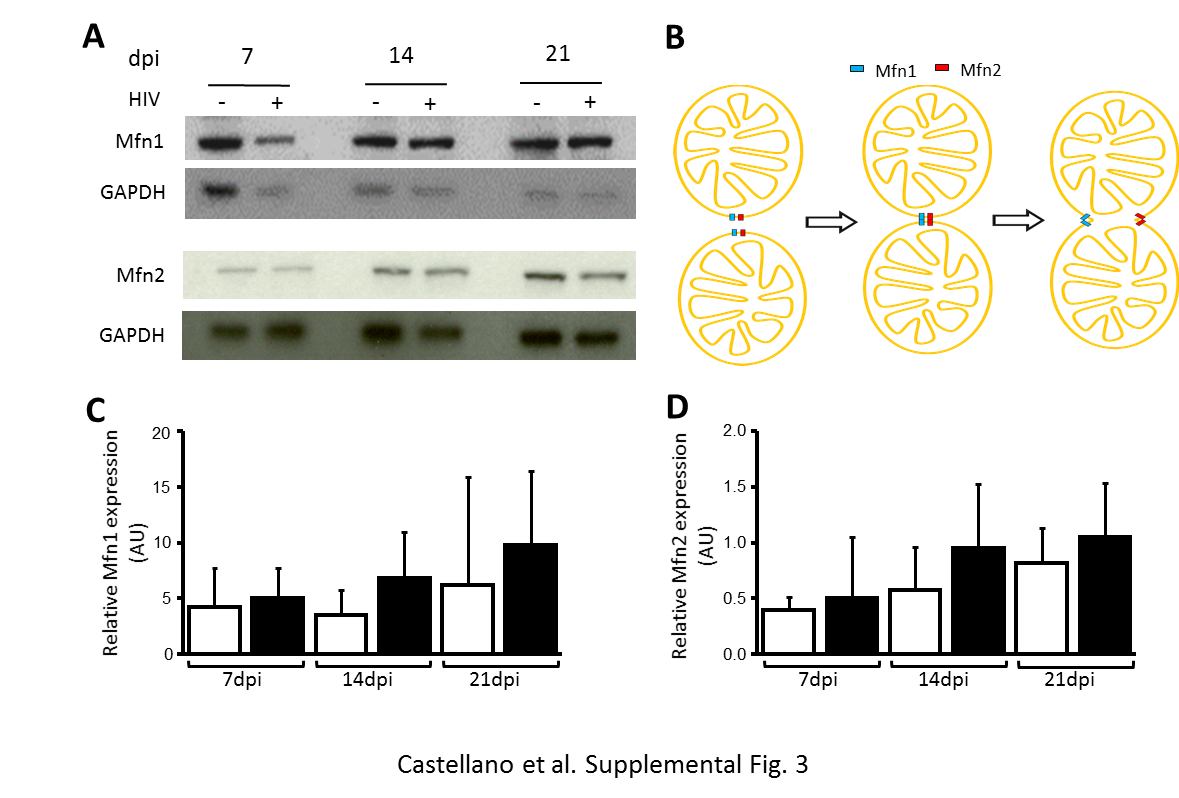
**

**
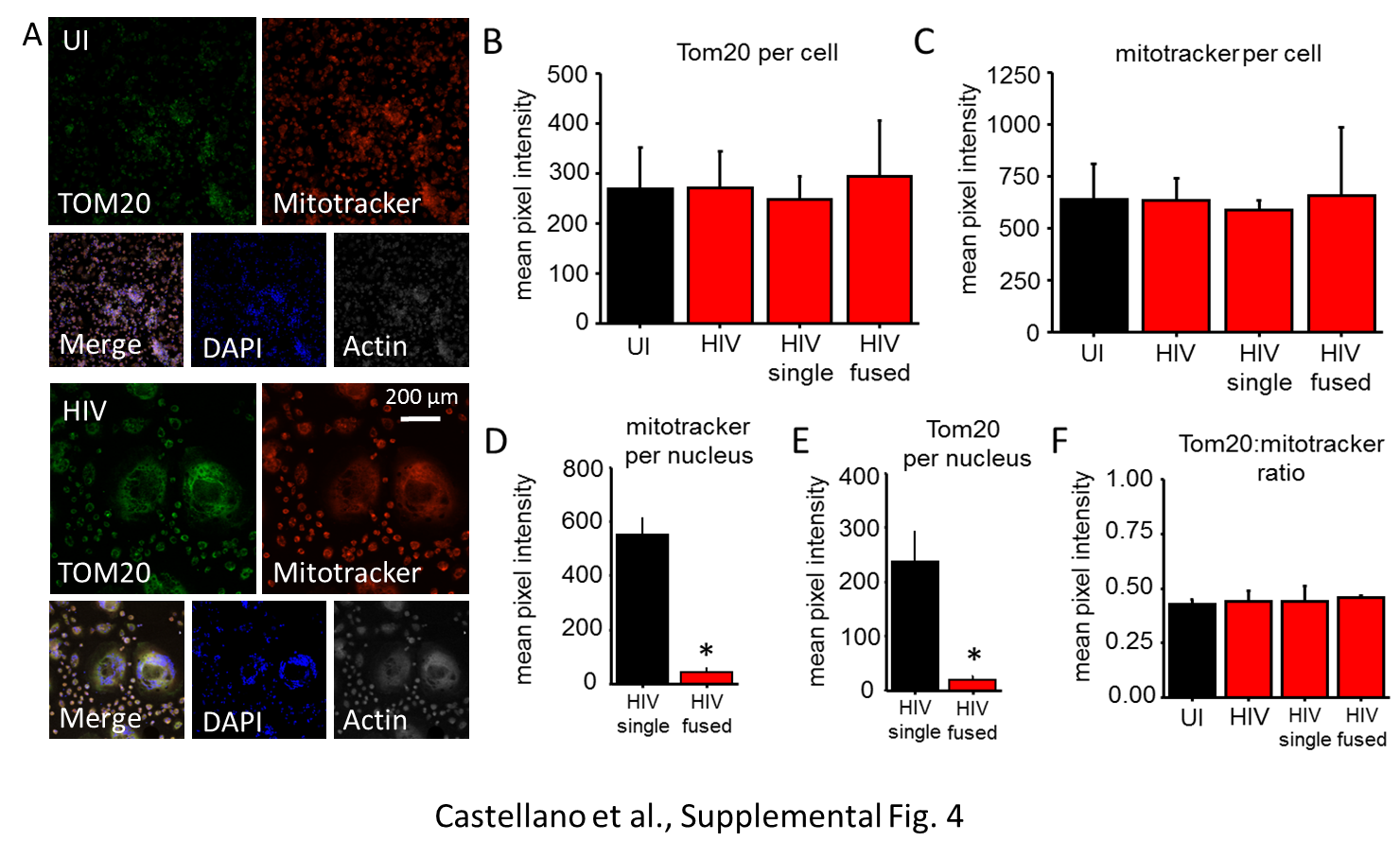
**

**
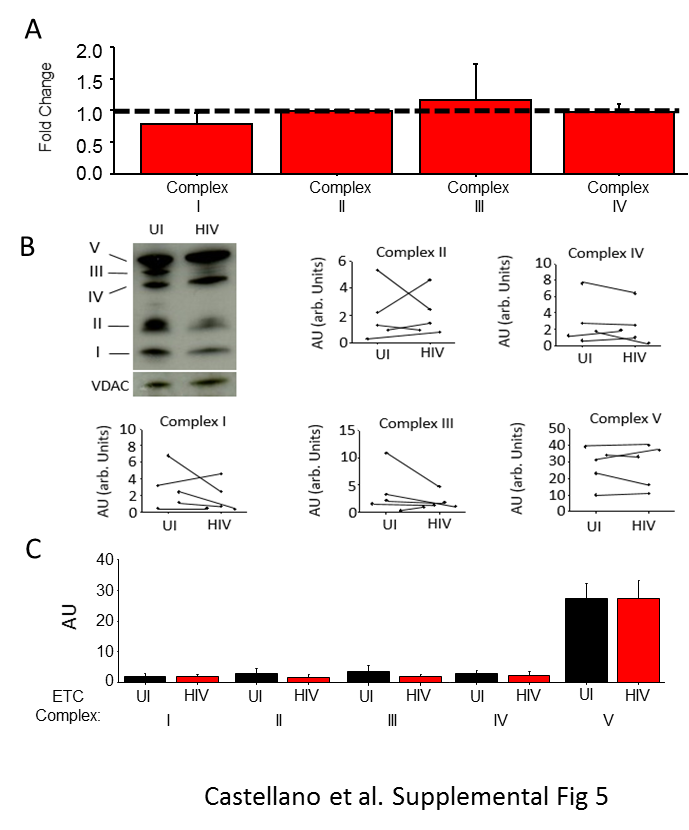
**
